# Protective NK Cell Reservoir Based on DNA Supramolecular Hydrogel for Enhanced Triple-Negative Breast Cancer Therapy

**DOI:** 10.34133/research.1436

**Published:** 2026-09-17

**Authors:** Wanxia Lu, Muyang Li, Wei He, Wanren Yang, Jinyue Fan, Haoxiang Chen, Chengyu Feng, Fengdi Yang, Chao Zhang, Qing Peng, Xiaolong Cao

**Affiliations:** ^1^ Department of Oncology, Zhujiang Hospital, Southern Medical University, Guangzhou 510282, P.R. China.; ^2^ Department of Hepatobiliary Surgery II, Zhujiang Hospital, Southern Medical University, Guangzhou 510282, P. R. China.; ^3^ Translational Medicine Research Center, Zhujiang Hospital, Southern Medical University, Guangzhou 510282, P.R. China.; ^4^ Central Laboratory of The Second Affiliated Hospital, School of Medicine, The Chinese University of Hong Kong, Shenzhen & Longgang District People’s Hospital of Shenzhen, Shenzhen, P.R. China.; ^5^ Department of Anesthesiology, Zhujiang Hospital, Southern Medical University, Guangzhou 510282, P.R. China.

## Abstract

Adoptive natural killer (NK) cell therapy represents a promising strategy for triple-negative breast cancer, but its therapeutic efficacy is often limited by poor intratumoral persistence and functional exhaustion within the immunosuppressive tumor microenvironment. Here, we developed an injectable DNA supramolecular hydrogel-based NK cell delivery platform (NK@DSH) that functions as a protective reservoir for transferred NK cells. The DNA supramolecular hydrogel enabled efficient NK cell encapsulation under mild conditions while preserving both cell viability and functionality. In vivo fluorescence tracking demonstrated prolonged intratumoral persistence of NK cells for up to 7 d and increased NK cell accumulation within tumor tissues. In a 4T1 tumor-bearing mouse model, NK@DSH produced stronger tumor growth inhibition than free NK cell administration while showing negligible systemic toxicity. This DSH-based strategy provides a generalizable platform for adoptive cell therapy, offering new insights into overcoming cell-persistence barriers in the treatment of solid tumors.

## Introduction

Breast cancer is the second most common cancer worldwide and the leading cause of cancer-related mortality among women [1–4]. Among the molecular subtypes of breast cancer, triple-negative breast cancer (TNBC) is recognized as one of the most aggressive forms, representing approximately 10% to 24% of all cases and remaining associated with a median overall survival of only 10.2 months for metastatic TNBC despite current therapeutic regimens [5,6]. Although targeted therapies and immunotherapies have recently expanded the treatment landscape, systemic management for many TNBC patients still relies heavily on cytotoxic chemotherapy due to the absence of clinically actionable receptor targets [1–3,7–10]. Unfortunately, the clinical utility of these cytotoxic treatments is frequently constrained by systemic toxicity, acquired resistance, and tumor recurrence, highlighting the urgent need for more effective therapeutic strategies for TNBC [2,5,6,11–14]. In recent years, adoptive natural killer (NK) cell therapy has emerged as a promising therapeutic strategy for TNBC due to its potent antitumor activity, favorable safety profile, and off-the-shelf applicability [6,15–19]. Nevertheless, the therapeutic efficacy of adoptively transferred NK cells against TNBC remains unsatisfactory, as the immunosuppressive tumor microenvironment (TME) severely restricts NK cell persistence and effector functions [18–27].

To address the functional exhaustion and poor retention of transplanted NK cells, numerous genetic and cellular engineering strategies have been devised to bolster the resilience of NK cells against TME-mediated immunosuppression [6,17,19,23–29]. Constitutive interleukin-15 (IL-15) expression and antioxidant protein overexpression are representative examples of such approaches and have been explored to enhance NK cell survival and cytotoxic activity [17,28,29]. Despite these incremental advances, such strategies only optimize the intrinsic cellular characteristics of NK cells. While these modifications confer short-term functional improvements, durable intratumoral retention and stable antitumor functionality are difficult to sustain within the persistent immunosuppressive TME [18,19,22–27]. Given that long-term in situ persistence of functional NK cells is the core prerequisite for sustained antitumor immune responses, biomaterial-based delivery systems have garnered extensive attention as a promising strategy to improve the intratumoral persistence of adoptively transferred NK cells [30–35].

Polymeric hydrogels, as 3-dimensional biomaterial networks with high water content and tunable properties, have been widely explored for cell encapsulation and localized delivery of therapeutic agents. Compared with conventional polymeric hydrogels, DNA supramolecular hydrogel (DSH) undergoes mild and rapid self-assembly under physiological conditions without harsh chemical or physical processing, which is favorable for encapsulating living cells with minimal disruption to their viability [35–42]. More importantly, the crosslinked architecture of DSH may help localized cell confinement and has the potential to reduce cellular exposure to soluble immunosuppressive cues [24–26,28,34,36]. Such properties suggest that DSH could serve as a peritumoral reservoir for NK cells, a feature that may help address the persistence and accumulation limitations commonly faced by adoptively transferred NK cells in solid tumors.

**Fig. 1.**
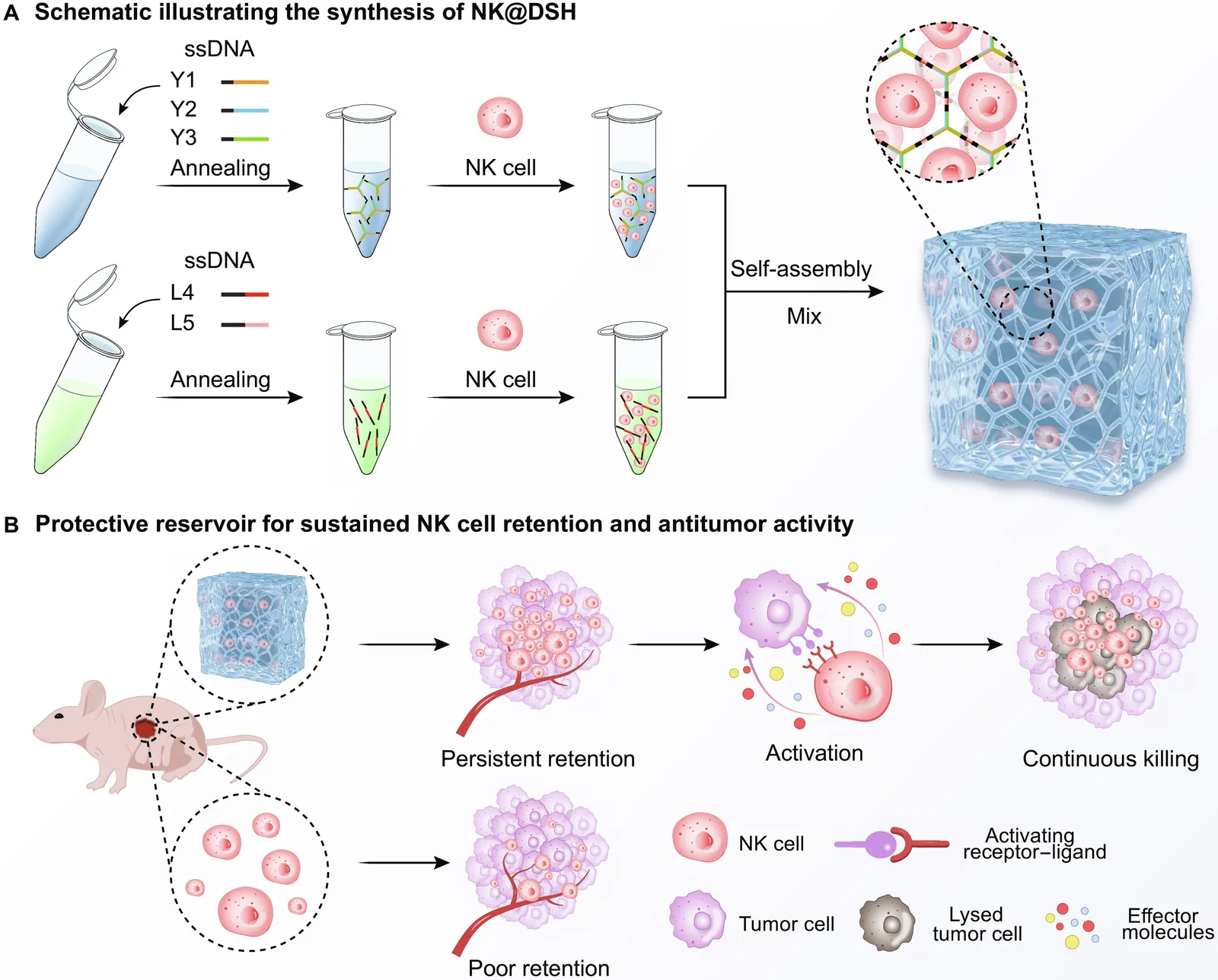
Schematic illustration of the design and therapeutic strategy of NK@DSH. (A) Schematic illustration of the fabrication of NK@DSH. Two sets of single-stranded DNA (ssDNA; Y1/Y2/Y3 and L4/L5) were separately annealed to generate Y-shaped and linear DNA building blocks. Following incubation with natural killer (NK) cells, the 2 DNA components were mixed to undergo supramolecular self-assembly, forming a 3-dimensional DNA hydrogel that encapsulates NK cells (NK@DSH). (B) Protective reservoir for sustained NK cell retention and antitumor activity. Following peritumoral administration, free NK cells exhibit limited local retention, resulting in unsustained cytotoxic activity. In contrast, the DNA supramolecular hydrogel functions as a protective peritumoral reservoir that prolongs local NK cell retention while preserving their viability and cytotoxic function. The sustained availability of functional NK cells enables continuous engagement with tumor cells, prolonged release of effector molecules, and persistent tumor cell killing.

In this study, we developed an injectable DSH-based NK cell delivery system (NK@DSH) that functions as a protective peritumoral reservoir for TNBC immunotherapy. As the foundation for this reservoir function, the DSH enabled efficient NK cell encapsulation under mild conditions while preserving both cell viability and functionality (Fig. 1A). In 4T1 tumor-bearing mice, prolonged retention and enhanced tumor infiltration of functionally active NK cells were achieved through DSH encapsulation compared with free NK cell administration. Furthermore, NK@DSH exhibited potent antitumor activity while maintaining negligible systemic toxicity. Collectively, this work highlights localized NK cell reservoir formation as an effective paradigm for improving NK cell retention and immunotherapeutic outcomes against TNBC (Fig. 1B).

## Results

### Preparation and characterization of the DNA hydrogel-based cell delivery

**Fig. 2.**
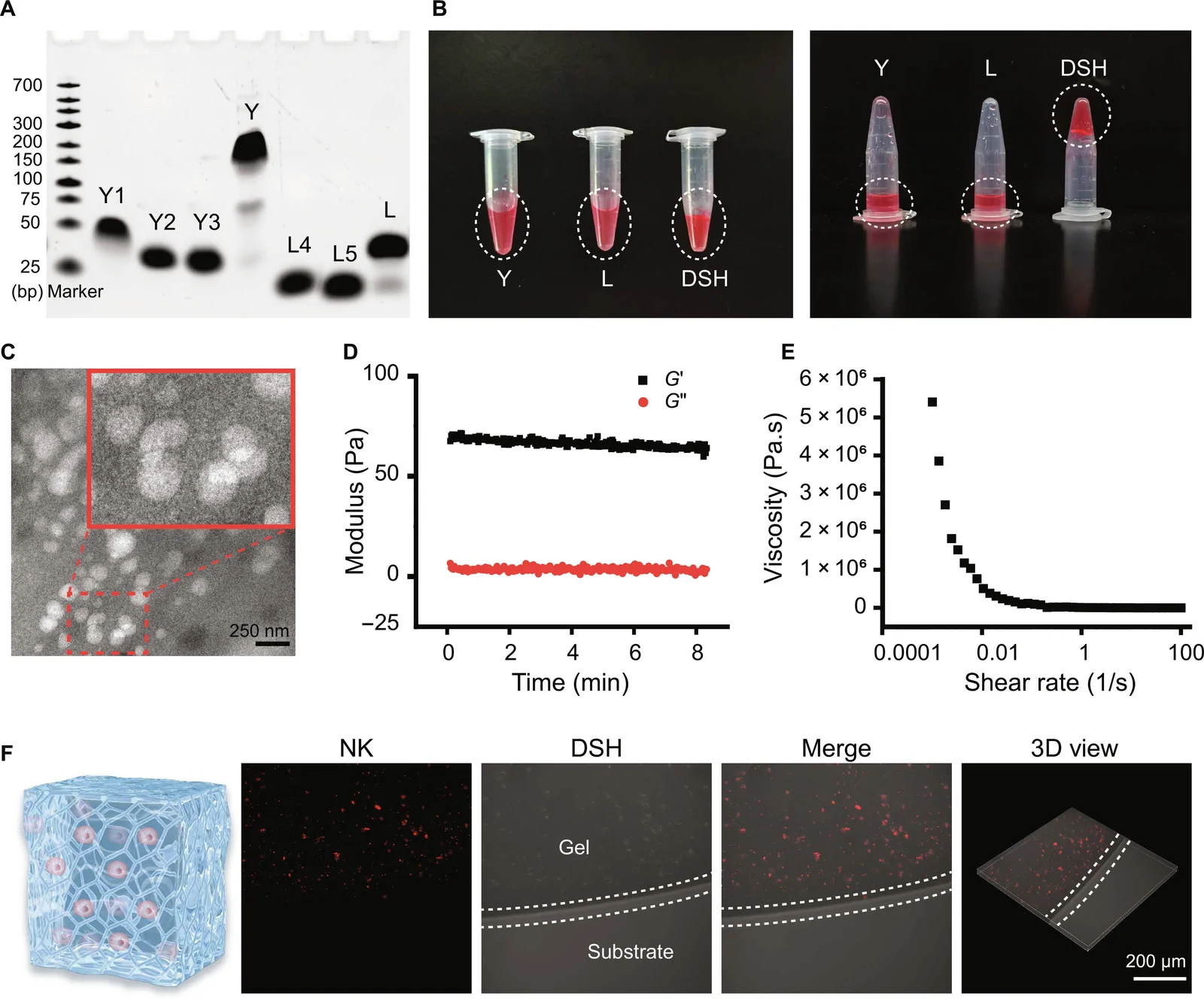
Synthesis and characterization of DNA supramolecular hydrogel (DSH) and NK@DSH. (A) Polyacrylamide gel electrophoresis analysis of single-stranded DNA, the Y-shaped scaffold (Y), and the L-linker (L). (B) Photographs depicting the Y and L solutions before combination and the resulting DSH after mixing. (C) Transmission electron microscopy image showing the porous microstructure of the DSH. (D) Time-sweep rheological analysis of the DSH showing the storage modulus (*G*′) and loss modulus (*G*″). (E) Steady-state shear viscosity of DSH. (F) Representative confocal images of NK@DSH showing the uniform distribution of mCherry-labeled natural killer (NK) cells (red) within the DSH. 3D, 3-dimensional.

The Y-scaffold (Y) and linear linker (L) served as the 2 primary building blocks to construct the DSH through the Watson–Crick base pairing principle [36–40]. Specifically, the Y-scaffold was assembled from 3 partially complementary single-stranded DNA (ssDNA) strands (Y1, Y2, and Y3), whereas the L-linker was formed by the hybridization of L4 and L5 (DNA sequences, Table S1). The successful assembly of the Y-scaffold and L-linker was validated via native polyacrylamide gel electrophoresis (PAGE), as evidenced by their slower migration rates compared to the constituent single strands (Fig. 2A). The DSH formed rapidly within seconds at room temperature upon mixing equal volumes of the Y-scaffold and L-linker solutions previously annealed from 95 to 4 °C, requiring no external stimuli such as ultraviolet light or temperature shifts, as shown in Fig. 2B. The formation of DSH was further confirmed by macroscopic observation, showing a distinct gel state after mixing the Y-scaffold and L-linker components (Fig. S1). A porous microstructure was observed within the DSH via transmission electron microscopy, which provides a favorable 3-dimensional scaffold for the accommodation and support of loaded NK cells (Fig. 2C). Next, the rheological properties of DSH were investigated. In the time-sweep test (Fig. 2D), the storage modulus (*G*′) was substantially higher than the loss modulus (*G*″), indicative of a well-defined viscoelastic network and confirming the successful formation of DSH. Additionally, DSH exhibited pronounced shear-thinning behavior (Fig. 2E), demonstrating its excellent injectability. This autonomous, stimulus-free gelation mechanism is essential for maintaining the structural integrity and biological activity of encapsulated NK cells [36–38,40,42]. For cell encapsulation, NK cells were premixed with the Y-scaffold and L-linker solutions independently prior to combining the 2 components. To evaluate whether cellular encapsulation affected the structural integrity of the hydrogel network, rheological measurements were further performed on NK@DSH. The *G*′ remained consistently higher than *G*″, and the overall viscoelastic behavior was comparable to that of DSH without NK cell loading (Fig. S2). These findings indicated that NK cell encapsulation did not compromise the mechanical stability of DSH. Importantly, the shear-thinning behavior was preserved after NK cell encapsulation, indicating that NK@DSH remained injectable despite cellular loading (Fig. S3). Furthermore, NK@DSH could be smoothly extruded through a syringe needle, demonstrating its favorable injectability for cell delivery applications (Fig. S4). Beyond the preserved rheological properties of NK@DSH, the spatial distribution of encapsulated NK cells within the hydrogel network was further characterized. Confocal laser scanning microscopy (CLSM) imaging revealed that NK cells were uniformly distributed throughout the DSH matrix (Fig. 2F).

### Effects of NK cell survival and activity encapsulated in DNA hydrogel in vitro

**Fig. 3.**
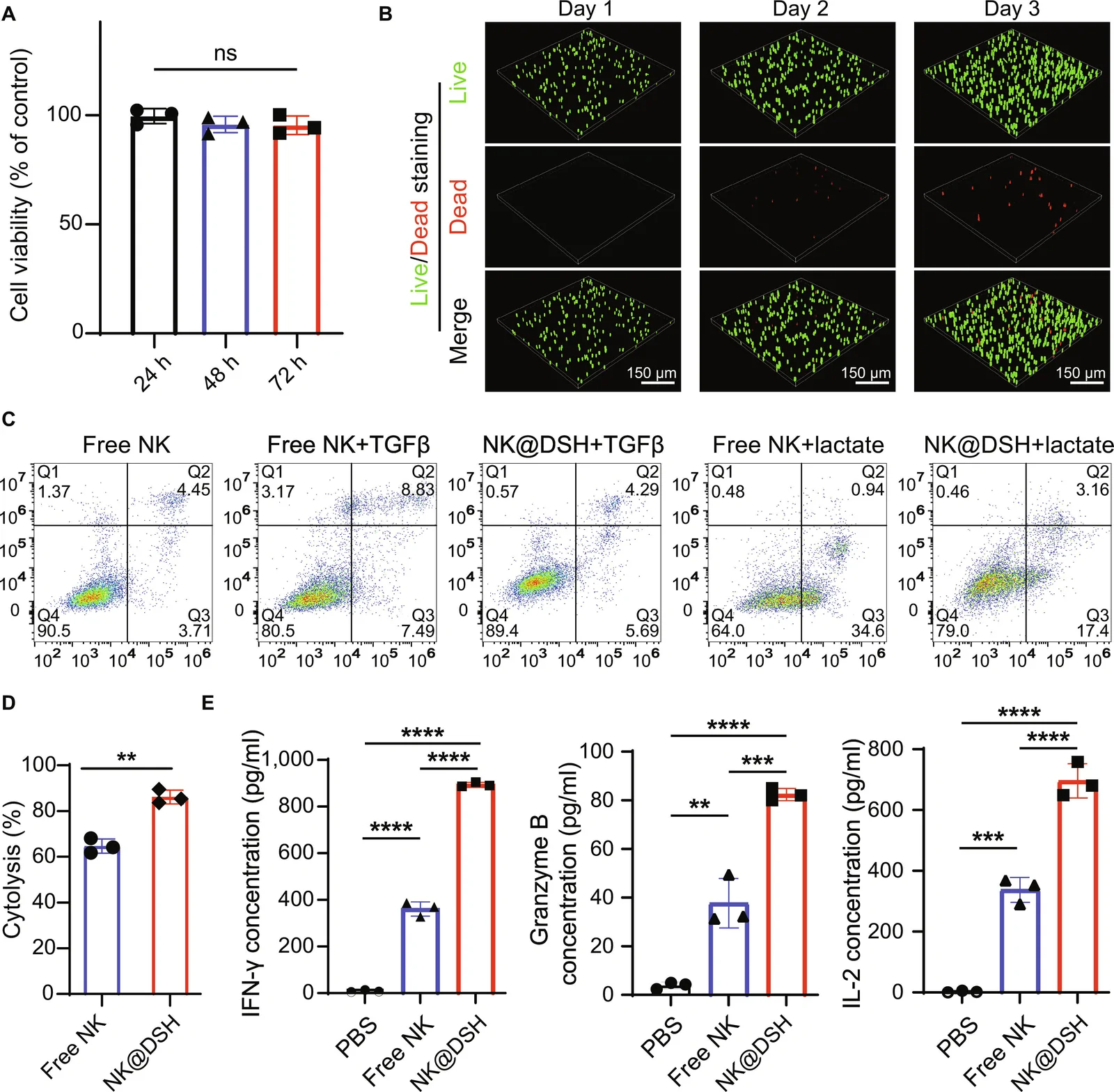
Effects of natural killer (NK) cell survival and activity encapsulated in DNA hydrogel in vitro. (A) In vitro biocompatibility assessment of DNA supramolecular hydrogel (DSH) on NK cells using Cell Counting Kit-8 assay (*n* = 3). (B) Fluorescence live/dead staining of NK cells encapsulated inside the DSH, imaged after 1, 2, and 3 d of incubation. (C) Representative Annexin V/propidium iodide flow cytometry plots of free NK cells and NK@DSH under normal or immunosuppressive conditions induced by transforming growth factor-β (TGFβ) or lactate. (D) Lactate dehydrogenase release assay showing the cytotoxicity of Free NK and NK@DSH against 4T1 cells (*n* = 3). (E) Enzyme-linked immunosorbent assay analysis of interferon-γ (IFN-γ), granzyme B, and interleukin-2 (IL-2) secretion by Free NK and NK@DSH following coculture with 4T1 cells (*n* = 3). ns, not significant; **P < 0.01, ***P < 0.001, ****P < 0.0001.

While NK cell therapy confers notable safety advantages and operates independently of prior antigen sensitization, its therapeutic efficacy remains constrained by poor cell persistence and TME-mediated functional exhaustion. Therefore, sustaining NK cell survival and preserving their antitumor cytotoxicity under hostile microenvironmental conditions are crucial to optimizing their therapeutic potential. To evaluate whether DSH could support NK cell survival and growth, we first assessed its biocompatibility. Cell viability remained above 96% following 72-h treatment with the DSH extract, confirming the nontoxic nature and excellent cytocompatibility of the hydrogel (Fig. 3A). Following encapsulation within the DSH, live/dead staining revealed a progressive increase in NK cell number over time, indicating that NK cells retained their capacity for sustained growth and proliferation within the hydrogel network (Fig. 3B). This effect is likely attributable to the porous architecture of the DSH network, which creates a favorable microenvironment for localized NK cell clustering, thereby supporting their viability and proliferative capacity. To further evaluate the protective effect of DSH under tumor-associated immunosuppressive conditions, NK cells were exposed to transforming growth factor-β (TGFβ) or lactate, followed by Annexin V/propidium iodide (PI) staining analysis. Compared with free NK cells, NK@DSH showed reduced apoptosis under both conditions, suggesting that DSH provided a protective microenvironment to maintain NK cell viability under immunosuppressive stress (Fig. 3C and Figs. S5 and S6). Next, the antitumor function of NK cells within the DSH was evaluated in vitro using 4T1 cells, a TNBC cell line. Cytotoxicity against 4T1 tumor cells was evaluated by quantifying lactate dehydrogenase (LDH) release after a 72-h coculture at an effector-to-target ratio of 1:2. Notably, the NK@DSH group exhibited enhanced cytolytic activity compared to the Free NK group (Fig. 3D). These results suggest that the protective microenvironment provided by DSH helps preserve NK cell viability under stressful conditions, thereby supporting sustained antitumor activity. Furthermore, the production of key effector molecules, including interferon-γ (IFN-γ), granzyme B, and IL-2, was augmented in the NK@DSH group compared with the Free NK control, suggesting that the hydrogel-encapsulated NK cells remained highly activated, thereby maintaining their capacity to effectively eliminate tumor cells even after a 72-h coculture (Fig. 3E). Collectively, these findings indicate that DSH encapsulation effectively preserves NK cell viability and cytolytic activity relative to unencapsulated cells, highlighting its potential to sustain antitumor efficacy within the immunosuppressive TME.

### NK cells encapsulated in the hydrogel persist in vivo

**Fig. 4.**
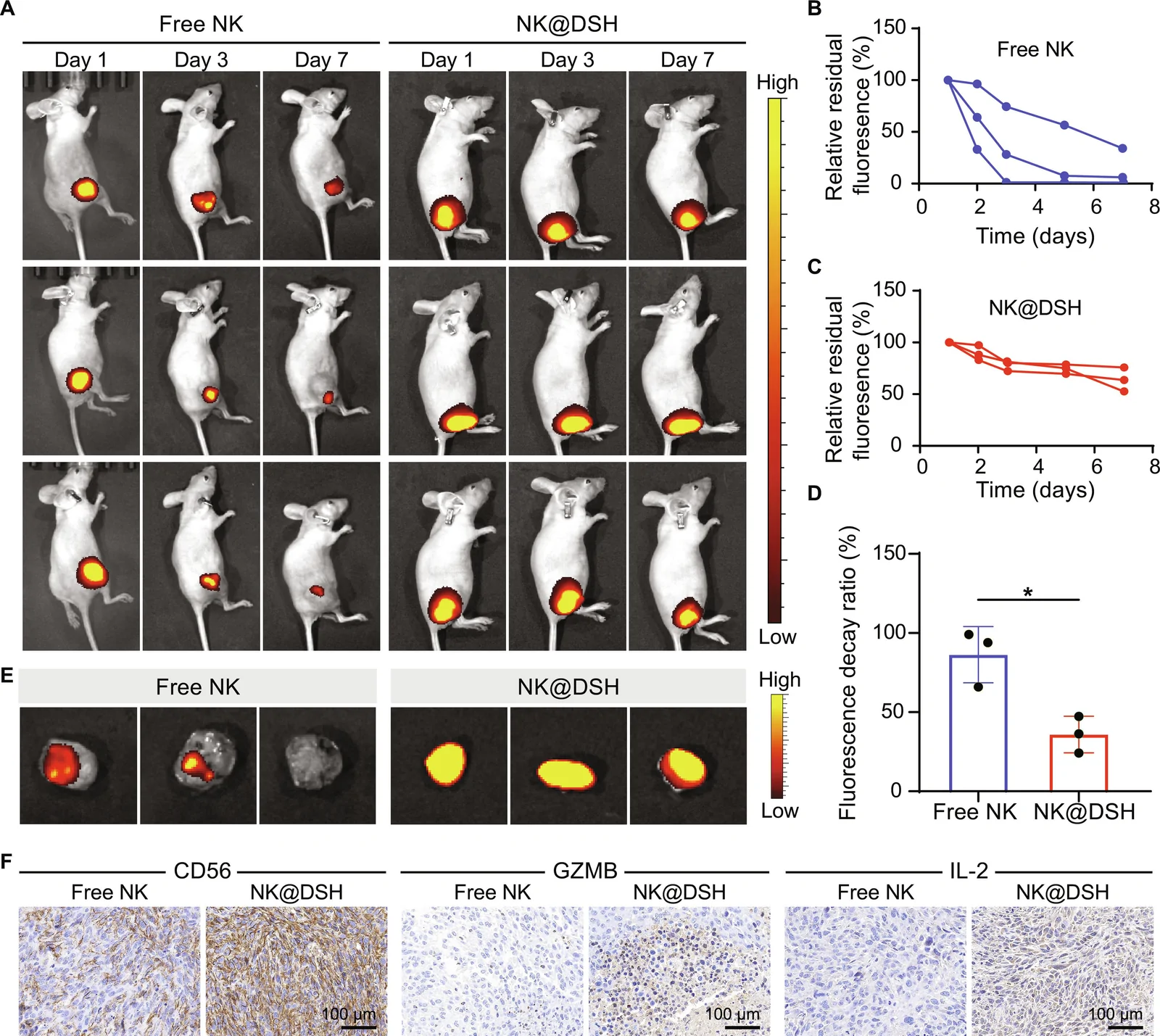
Natural killer (NK) cells encapsulated in the hydrogel persist in vivo. (A) Fluorescence images tracking the in vivo retention of indocyanine green (ICG)-labeled Free NK and NK@DSH postperitumoral injection. (B and C) Normalized fluorescence intensity of Free NK and NK@DSH groups over time, relative to day 1. (D) Fluorescence signal decay on day 7 in the Free NK and NK@DSH groups. (E) Representative ex vivo fluorescence images of excised tumors. (F) Representative immunohistochemical staining of human CD56, granzyme B (GZMB), and interleukin-2 (IL-2) in tumor slices. *n* = 3, *P < 0.05.

To investigate whether the DSH could exert a protective effect to improve the retention and persistence of NK cells in vivo, we first employed an IVIS Spectrum imaging system to track the fluorescence signals at various time points following the peritumoral administration of NK cells, which were encapsulated within the DSH and labeled with indocyanine green (ICG). As shown in Fig. 4A, the fluorescence signal of the Free NK group rapidly declined over time, whereas that of the NK@DSH group remained strong for up to 7 d. Quantitative analysis of the normalized fluorescence intensity over time further demonstrated a sustained retention of signal in the NK@DSH group compared with Free NK (Fig. 4B and C). At day 7, the decrease in fluorescence intensity in the Free NK group was 2.4-fold greater than that observed in the NK@DSH group, indicating a markedly attenuated signal loss in the hydrogel-encapsulated NK cells (Fig. 4D). To verify the intratumoral retention of NK cells, tumors were collected on day 7 after euthanasia and subjected to ex vivo fluorescence imaging (Fig. 4E). Consistent with the in vivo observations, the ex vivo imaging of the harvested tumors revealed a stronger fluorescence signal in the NK@DSH group, whereas the signal in the Free NK group was markedly diminished (Fig. 4E). In addition, tumor sections were subjected to immunohistochemical (IHC) staining with an anti-human CD56 antibody to further investigate the intratumoral distribution of NK cells. Notably, tumors treated with NK@DSH exhibited an increased density of CD56-positive cells compared to the Free NK group, confirming a higher abundance of retained NK cells within tumors (Fig. 4F and Fig. S7), which may be associated with the supportive local microenvironment provided by the DSH, which helps maintain NK cell retention and effector activity. Consistent with the robust cell infiltration, the tumor tissues of the NK@DSH group displayed markedly elevated levels of granzyme B, IL-2, and IFN-γ compared to the Free NK group (Fig. 4F and Figs. S8 to 10), demonstrating sustained local immune activation and potent effector functions in vivo. Collectively, these data confirm that the DSH serves as a supportive reservoir that effectively prolongs the retention and drives the active tumor infiltration of NK cells while sustaining their cytotoxic effector functions in vivo.

### Enhanced antitumor activity of NK@DSH in vivo

**Fig. 5.**
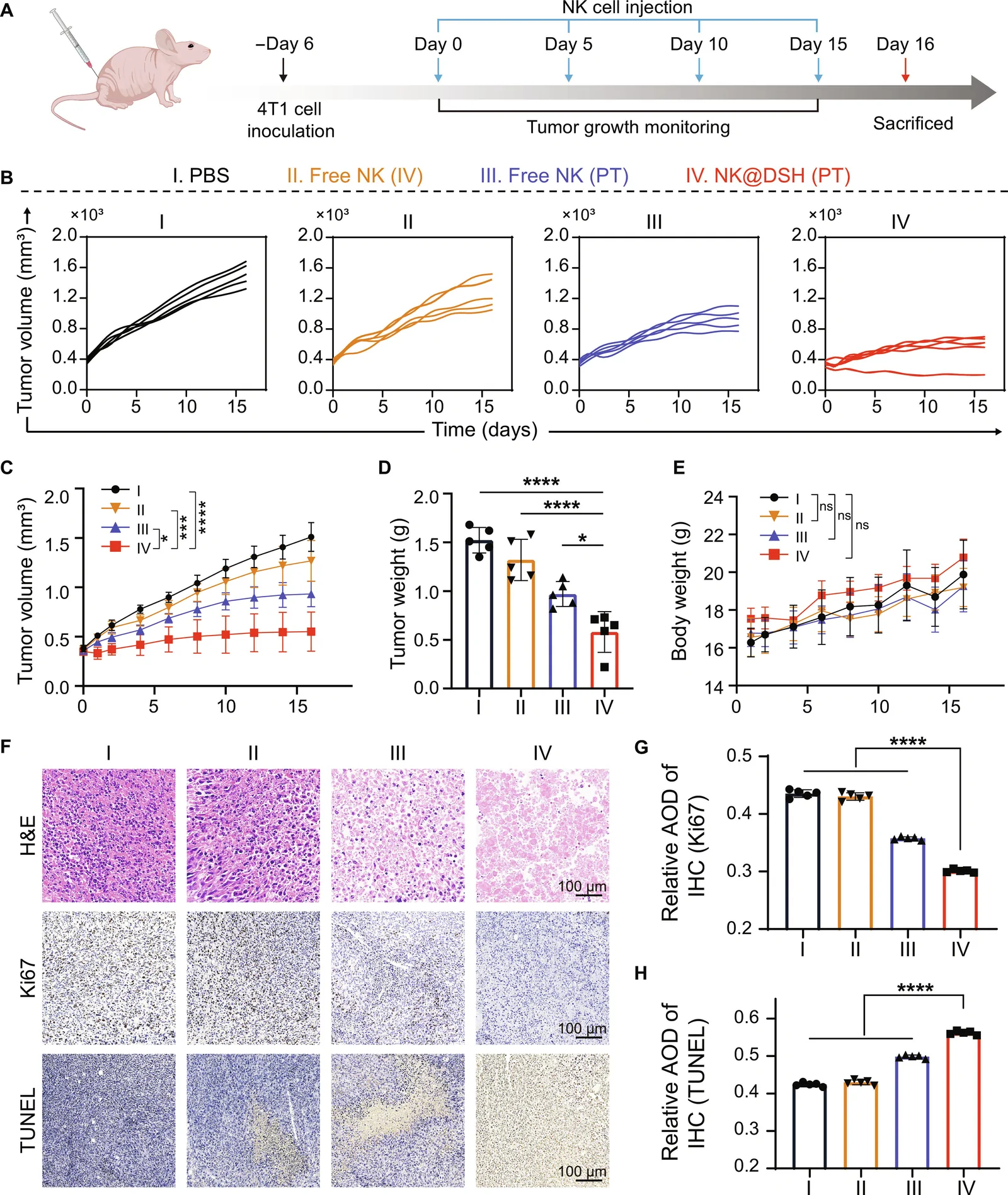
Enhanced antitumor activity of NK@DSH in vivo. (A) Schematic illustration of the treatment schedule and experimental procedure in the 4T1 tumor-bearing mouse model. 4T1 tumor-bearing mice received natural killer (NK) cell administrations on days 0, 5, 10, and 15 after tumor inoculation and were sacrificed on day 16 for subsequent analysis. (B) Individual tumor volume measurements over time following treatment with phosphate-buffered saline (PBS), Free NK (IV), Free NK (PT), or NK@DSH (PT). (C) Time-dependent changes in tumor volume following the indicated treatments. (D) Weights of excised 4T1 tumors harvested on day 16. (E) Changes in body weight. (F) Representative hematoxylin and eosin (H&E), Ki67, and TUNEL (terminal deoxynucleotidyl transferase–mediated deoxyuridine triphosphate nick end labeling) staining images of excised tumor sections. Scale bar, 100 μm. (G and H) Quantification of cell proliferation (Ki67) and apoptosis (TUNEL) in tumor slices. *n* = 5, *P < 0.05, ***P < 0.001, ****P < 0.0001. ns, not significant.

Given that DSH encapsulation markedly enhanced the local retention of NK cells in vivo, we next sought to determine whether the advantages conferred by this specific delivery strategy could further improve the therapeutic performance of NK cell-based immunotherapy against TNBC. To this end, 6 d after 4T1 tumor cell inoculation, BALB/c nude mice were randomly assigned to 4 groups: phosphate-buffered saline (PBS), free NK cells administered intravenously (Free NK [IV]), free NK cells administered via peritumoral injection (Free NK [PT]), and DSH-encapsulated NK cells administered via peritumoral injection (NK@DSH [PT]). Tumor progression was subsequently monitored throughout the treatment period (Fig. 5A). As shown in Fig. 5B and C, both the PBS and Free NK (IV) groups showed negligible antitumor efficacy. Although Free NK (PT) administration led to moderate tumor suppression, the NK@DSH (PT) treatment yielded the most robust growth inhibition. On day 16 posttreatment, the NK@DSH (PT) group exhibited the lowest tumor burden, as substantiated by the lowest average tumor weight (Fig. 5D and Fig. S11). No appreciable changes in body weight were detected across the 4 treatment groups during the experimental period (Fig. 5E), suggesting that NK@DSH treatment was well tolerated in vivo. Histological analyses of excised tumor tissues provided mechanistic insight into the observed therapeutic effects. Compared with the other groups, tumors treated with NK@DSH (PT) exhibited more extensive tissue damage, increased apoptotic cell death, and reduced proliferative activity, as evidenced by hematoxylin and eosin (H&E), TUNEL (terminal deoxynucleotidyl transferase–mediated deoxyuridine triphosphate nick end labeling), and Ki67 staining, respectively (Fig. 5F to H). These findings, together with the enhanced retention, persistence, and local accumulation of NK cells observed previously, indicate that DSH encapsulation confers durable therapeutic efficacy against TNBC in vivo.

### Biocompatibility and biosafety evaluation of the NK@DSH

**Fig. 6.**
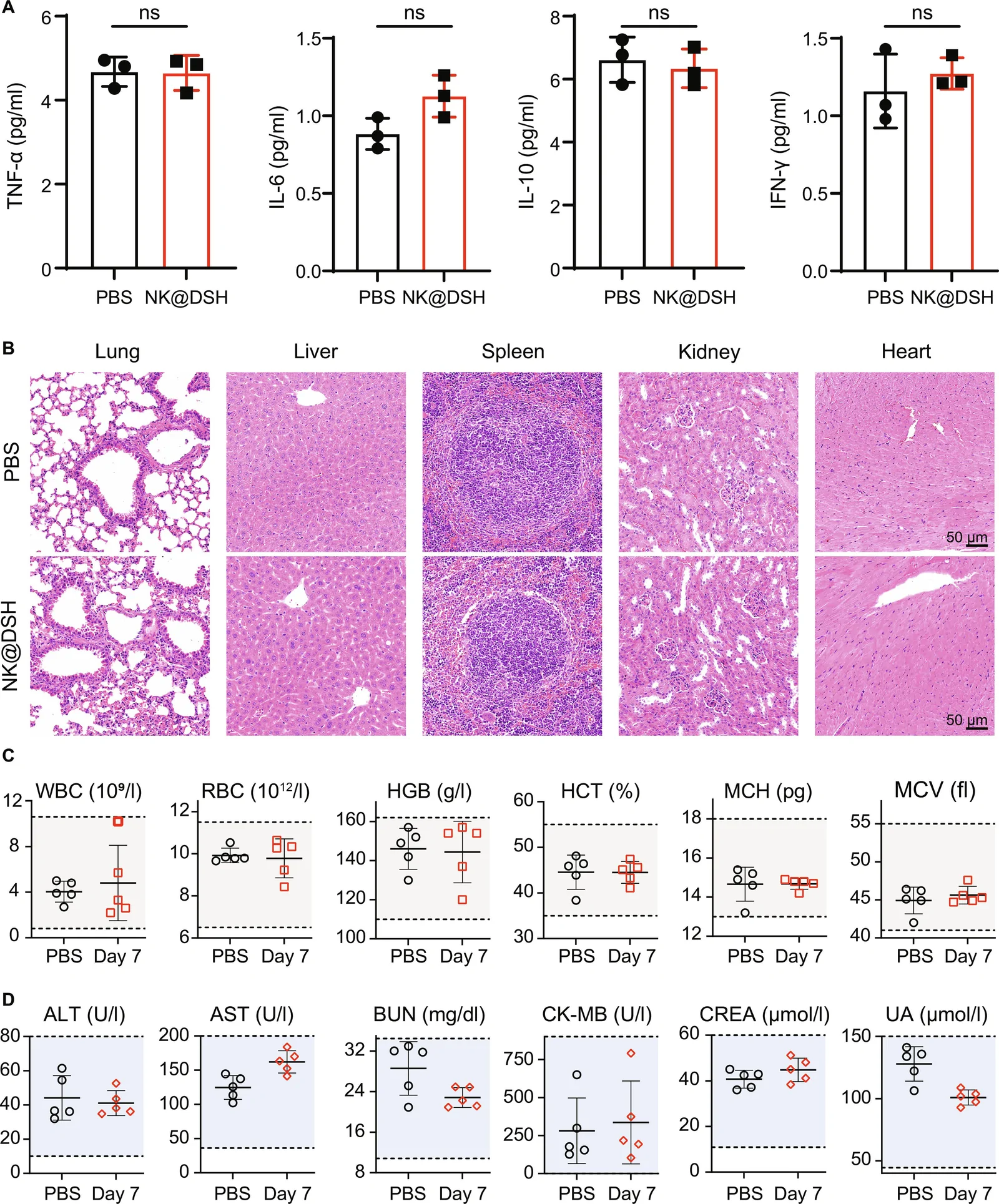
Biocompatibility and biosafety evaluation of the NK@DSH. (A) Enzyme-linked immunosorbent assay analysis of serum levels of interferon-γ (IFN-γ), interleukin-6 (IL-6), IL-10, and tumor necrosis factor-α (TNF-α) in healthy mice treated with the phosphate-buffered saline (PBS) or NK@DSH (PT) (*n* = 3). (B) Representative histological images of the heart, liver, spleen, lung, and kidney following hematoxylin and eosin staining in the PBS and NK@DSH (PT) groups. Scale bars, 50 μm. (C) Routine hematological analysis on day 7 (*n* = 5). (D) Serum biochemical analysis on day 7 (*n* = 5). ns, not significant; WBC, white blood cell; RBC, red blood cell; HGB, hemoglobin; HCT, hematocrit; MCH, mean corpuscular hemoglobin; MCV, mean corpuscular volume; ALT, alanine transaminase; AST, aspartate transaminase; BUN, blood urea nitrogen; CK-MB, creatine kinase-MB; CREA, creatinine; UA, uric acid.

While DSH-mediated delivery markedly enhanced NK cell retention and antitumor efficacy, the translational potential of this strategy is contingent upon whether these therapeutic benefits can be realized without eliciting adverse biological effects [31–35]. Therefore, the biosafety profile of NK@DSH (PT) was comprehensively investigated. As shown in Fig. 6A, circulating IFN-γ, tumor necrosis factor–α (TNF-α), IL-6, and IL-10 exhibited comparable levels in the PBS and NK@DSH (PT) groups, indicating that prolonged NK cell retention mediated by DSH did not provoke excessive systemic immune activation, inflammatory responses, or cytokine release syndrome. Furthermore, no obvious histopathological abnormalities were detected in H&E-stained sections of key visceral organs (heart, liver, spleen, lungs, and kidneys) in the NK@DSH (PT) group relative to the PBS group at day 7 posttreatment (Fig. 6B). Consistently, routine hematological parameters, such as leukocyte count (white blood cell), erythrocyte count (red blood cell), hemoglobin, hematocrit, mean corpuscular hemoglobin, and mean corpuscular volume, remained stable within normal physiological limits following NK@DSH (PT) administration, indicating the absence of detectable hematological toxicity (Fig. 6C). Additionally, serum biochemical analyses revealed no evidence of treatment-associated organ dysfunction, further supporting the favorable biosafety profile of NK@DSH (PT) (Fig. 6D). Collectively, these findings demonstrate that DSH-mediated NK cell delivery exhibits excellent biocompatibility and negligible systemic toxicity in vivo.

## Conclusion

In this study, a DSH-based NK cell delivery platform (NK@DSH) was developed as a protective peritumoral reservoir to prolong the retention and maintain the functionality of NK cells for durable antitumor efficacy. We identified that encapsulated NK cells achieved survival and robust proliferation, presumably facilitated by localized cell-to-cell contact and the efficient diffusion of nutrients and oxygen within the porous DSH network. Consistent with the proposed reservoir effect, in vivo fluorescence tracking revealed that DSH encapsulation retained NK cells at the treatment site for up to 7 d and promoted their subsequent infiltration into tumor tissues. The prolonged local retention of NK cells may be attributed to the protective function of DSH under immunosuppressive tumor conditions. Flow cytometric analysis revealed that DSH encapsulation reduced NK cell apoptosis induced by TGFβ and lactate stimulation, suggesting that DSH preserves NK cell survival by mitigating tumor-associated suppressive stresses. Importantly, consistent increases in IFN-γ, granzyme B, and IL-2 production across both the in vitro and in vivo experiments indicate that NK cell effector functions were preserved following DSH encapsulation. Given that the immunosuppressive TME of TNBC is a major driver of NK cell dysfunction, these findings support the notion that the DSH reservoir helps mitigate its detrimental impact on transferred NK cells [11,18–27]. By providing a protective niche that sustains NK cell survival and functionality under immunosuppressive conditions, the DSH reservoir enables prolonged local persistence of functional NK cells, thereby contributing to the enhanced cytotoxicity and antitumor efficacy of NK@DSH therapy compared with free NK cells.

The NK@DSH platform offers a generalizable strategy for adoptive cell therapy that could be extended to other immune effector cells, particularly in solid tumors where insufficient cell persistence remains a major translational barrier [6,19,23,31–33,35]. Moreover, the modular nature of DSH provides ample opportunities for further optimization of this delivery platform [36–49]. In this regard, the duration and controllability of NK cell release from the DSH network could be improved by incorporating responsive elements that allow hydrogel degradation kinetics to be tuned according to the therapeutic window required for sustained NK cell activity.

## Materials and Methods

### Reagents

Dulbecco’s modified Eagle’s medium, fetal bovine serum (FBS), and penicillin–streptomycin were purchased from Gibco (Waltham, MA, USA), and X-VIVO 15 serum-free medium was obtained from LONZA (Basel, Switzerland). Recombinant human IL-2 and 2-mercaptoethanol were obtained from Tianjin Saiensi Biotechnology Co., Ltd. (Tianjin, China) and Sigma-Aldrich (Darmstadt, Germany), respectively. Cell Counting Kit-8 (CCK-8) reagent and Annexin V-fluorescein isothiocyanate (FITC)/PI apoptosis detection reagent were purchased from Aladdin Reagent (Shanghai, China). Calcein-AM/PI staining reagent and the LDH cytotoxicity assay kit with WST-8 were obtained from Beyotime Biotechnology (Shanghai, China). Enzyme-linked immunosorbent assay (ELISA) kits for TNF-α, IL-6, IFN-γ, and IL-10 were purchased from Elabscience (Wuhan, China), and ICG-*N*-hydroxysuccinimide (NHS) was obtained from Ruixi Biotech. DNA oligonucleotides were custom-synthesized by Sangon Biotech (Shanghai, China) and used as received.

### Cell culture

Murine 4T1 breast cancer cells (Procell, Wuhan, China) were routinely maintained in Dulbecco’s modified Eagle’s medium containing 10% FBS and 1% penicillin–streptomycin under standard culture conditions (37 °C, 5% CO₂). Human NK-92MI cells were purchased from Procell Life Science & Technology Co., Ltd. (Wuhan, China) and grown in X-VIVO 15 medium containing 10% FBS, IL-2, and 2-mercaptoethanol under the same culture conditions.

### Synthesis of DSH and NK@DSH

Y1-Y3 and L4/L5 oligonucleotides (2 mM each) were separately dissolved in PBS containing 2 mM MgCl_2_ (pH 7.2). Y- and L-building blocks were formed by programmed annealing from 95 to 4 °C at 1 °C/min. For DSH preparation, equal volumes of annealed Y-scaffold and L-linker solutions were mixed at room temperature, where complementary DNA hybridization drove rapid network formation. For NK@DSH fabrication, freshly isolated NK cells were resuspended at 1 × 10^6^ cells/ml and separately mixed with the Y-scaffold and L-linker solutions. Following mixing of the Y-scaffold and L-linker solutions with the NK cell suspension, the final concentrations of Y1, Y2, and Y3 were 250 nM each, whereas those of L4 and L5 were 375 nM each in the resulting NK@DSH hydrogel. The 2 cell-loaded precursor solutions were then combined, yielding NK@DSH within seconds under ambient conditions.

### Native PAGE

The assembly of DNA building blocks was analyzed by native PAGE. Y-scaffold, L-linker, and DSH precursor mixtures were prepared in PBS containing 2 mM MgCl_2_ and mixed with native loading buffer. Samples were loaded onto a native polyacrylamide gel and electrophoresed in 1× tris-acetate-EDTA buffer at constant voltage. After electrophoresis, DNA bands were visualized using a nucleic acid staining reagent and imaged with a gel documentation system.

### Transmission electron microscopy

Freshly prepared DSH was diluted with PBS and deposited onto a carbon-supported copper grid. After adsorption, excess liquid was removed with filter paper, and the grid was negatively stained with 2% uranyl acetate or phosphotungstic acid. The dried samples were examined by transmission electron microscopy under ambient conditions.

### Rheological experiments

Rheological properties of DSH were measured using a rheometer equipped with a 25-mm parallel-plate geometry. Time-sweep measurements were performed at 25 °C with the oscillation frequency and strain set to 1 Hz and 1%, respectively, and data were collected every 2 s. Shear-thinning behavior was characterized by flow-curves measurements over a shear-rate range of 0.001 to 100 s^−1^.

### NK cell encapsulation via CLSM

To visualize NK cell distribution within the hydrogel matrix, mCherry-expressing NK cells were encapsulated during DSH gelation. The resulting NK@DSH was examined by CLSM (Nikon, Japan), and Z-stack scanning was used to reconstruct the 3-dimensional arrangement of the encapsulated cells.

### Cytotoxicity test of DSH

DSH cytocompatibility toward NK cells was evaluated using a CCK-8 assay. NK cells were cultured in 96-well plates at 2 × 10^3^ cells per well in 100 μl of complete medium. After overnight culture, the plates were centrifuged, and the culture medium was exchanged with 100 μl of fresh medium containing 10 μl of DSH extract; wells receiving the same volume of PBS served as controls. CCK-8 reagent (10%) was added to each well, and the mixture was incubated in the dark for 25 min at 24, 48, and 72 h, respectively, before absorbance was measured.

### Cell survival in DSH

Cell survival and spatial distribution inside DSH were assessed by live/dead staining. At 1, 2, and 3 d of coculture, the inserts were removed, followed by gentle PBS washing. Samples were stained with Calcein-AM/PI at 37 °C for 1 h in the dark. CLSM imaging at excitation wavelengths of 488 and 568 nm was used to visualize live and dead cells, respectively.

### Flow cytometric analysis of NK cell apoptosis

To evaluate the protective effect of DSH under TME-relevant suppressive stress, NK-92MI cells (7 × 10^5^ cells per well) were allocated to 5 groups: untreated Free NK control, Free NK treated with TGFβ, NK@DSH treated with TGFβ, Free NK treated with lactate, and NK@DSH treated with lactate. TGFβ and lactate were added to the culture medium at final concentrations of 10 ng/ml and 7.5 mM, respectively. In the NK@DSH groups, the same number of NK cells was encapsulated in DSH as described above. Following a 72-h incubation, the cells were pelleted, washed with PBS, and labeled with Annexin V-FITC and PI using the commercial apoptosis detection kit. Fluorescence signals were acquired by flow cytometry, and the distribution of viable, apoptotic, and membrane-compromised NK cells was determined from the Annexin V-FITC/PI profiles.

### In vitro cytotoxicity assay

NK cell cytotoxicity against 4T1 target cells was quantified using an LDH release assay kit containing WST-8. After overnight adherence of 4T1 cells plated in 96-well plates, cells were treated with PBS, free NK cells, or NK@DSH at an effector-to-target ratio of 1:2. The coculture was maintained for 72 h. Untreated 4T1 cells served as the spontaneous LDH release control, whereas cells exposed to LDH release reagent were used as the maximum release control. Cell-free background controls were included for each medium or treatment condition.

After incubation, supernatants were collected and mixed with LDH detection solution. The absorbance at 450 nm was measured after the reaction was stopped with the stop solution. The calculation formula is as follows:

$$\begin{array}{c} \text{Cytotoxicity}\;\left(\%\right)=\frac{{\mathrm{OD}}_{\text{sample}}-{\mathrm{OD}}_{\text{untreated control}}}{{\mathrm{OD}}_{\text{maximum}\;\mathrm{LDH}\;\text{release}}-{\mathrm{OD}}_{\text{untreated control}}}\times 100\% \end{array}$$ (1)

### ELISA for cytokine detection

IL-2, granzyme B, and IFN-γ concentrations in culture supernatants were quantified using commercial ELISA kits. Absorbance was recorded at 450 nm after color development.

### In vivo antitumor study

For in vivo studies, 4- to 6-week-old female BALB/c nude mice (supplied by Best-Test Bio-Tec, Zhuhai, China) were utilized under a protocol approved by the Southern Medical University Ethics Committee (No. LAEC-2026-019) in compliance with the animal care guidelines of Guangdong Province. The animal facility maintained standard environmental conditions, including a temperature of 22 °C and relative humidity of 50%. Mice were housed in standard cages throughout the experimental period. A subcutaneous 4T1 tumor model was established by inoculating 5 × 10^6^ cells into the unilateral inguinal region. When tumor volumes reached approximately 200 mm^3^, mice were assigned to 4 groups (*n* = 5 per group): PBS (peritumoral injection of PBS), Free NK (PT) (peritumoral injection of free NK cells), Free NK (IV) (tail vein injection of free NK cells), and NK@DSH (PT) (peritumoral injection of NK@DSH). For peritumoral administration, NK@DSH was prepared prior to administration and loaded into a syringe after gel formation. The hydrogel was then administered via peritumoral injection using a 29G needle.

Formulations containing NK cells were administered on days 0, 5, 10, and 15, with each mouse receiving an equivalent dose of approximately 2 × 10^6^ NK cells per administration. Tumor length and width were measured every 2 d with a digital caliper, and tumor volume was calculated using the formula *V* = (*L* × *W*^2^)/2, where *L* and *W* represent the longest diameter and perpendicular width, respectively. Mouse body weight was recorded at the same time points. On day 16, mice were euthanized, and tumor tissues were collected for downstream analysis. Tumor sections were subjected to H&E, TUNEL, and Ki-67 IHC staining, followed by imaging with a digital slide scanner.

### In vivo fluorescence imaging and IHC analysis of tumor-infiltrating NK cells

NK cells were labeled with ICG-NHS before encapsulation. ICG-NHS contains an NHS ester group that reacts with amino groups on cell-surface proteins, enabling covalent conjugation of the near-infrared fluorescent dye to NK cells for in vivo fluorescence tracking. Whole-body fluorescence signals were acquired using an IVIS Spectrum imaging system (PerkinElmer, USA) with excitation and emission wavelengths of 780 and 830 nm, respectively, on days 1, 3, and 7 after administration. Fluorescence intensity was quantified with Living Image software.

To specifically evaluate the infiltration and functional status of transferred human NK-92MI cells within the mouse 4T1 TME, tumor sections were subjected to IHC staining for CD56, granzyme B, and IL-2 using human-specific antibodies. Specifically, rabbit anti-human CD56 antibody (Abcam, ab220360), rabbit anti-human granzyme B antibody (Abcam, ab4059), and mouse anti-human IL-2 antibody (R&D Systems, MAB602) were used for IHC staining.

### Histological and biosafety examination

Biosafety of the DSH carrier was evaluated in a separate cohort of mice receiving NK@DSH injections, with PBS-treated mice used as controls (*n* = 5 per group). At the end point, blood was collected for hematological profiling, serum biochemistry, and cytokine quantification. Serum IFN-γ, TNF-α, IL-6, and IL-10 concentrations were measured by ELISA. After fixing the kidneys, heart, liver, lungs, and spleen in 4% paraformaldehyde, they were sectioned and stained for pathological assessment.

### Statistical analysis

Data are presented as means ± SD. Each experiment included at least 3 replicates unless otherwise stated. Differences between 2 groups were analyzed using Student *t* test. Statistical significance was defined as follows: ns, not significant; *P < 0.05, **P < 0.01, ***P < 0.001, and ****P < 0.0001. Data analysis and graph generation were performed in GraphPad Prism 9.

## Data Availability

All data generated or analyzed during this study are included in this article and its supplementary files.
