# Supplementary material for: Protective NK Cell Reservoir Based on DNA Supramolecular Hydrogel for Enhanced Triple-Negative Breast Cancer Therapy
Source: Research (Wash D C). 2026 Sep 17;9:1436. doi: 10.34133/research.1436 (PMC13583129; doi:10.34133/research.1436)
Supplement: Supplementary file 1 [file research.1436.f1.docx]

**Supplementary Information**

**Protective NK Cell Reservoir Based on DNA Supramolecular Hydrogel for Enhanced Triple-Negative Breast Cancer Therapy**

Wanxia Lu^1†^, Muyang Li^1†^, Wei He^1†^, Wanren Yang^5^, Jinyue Fan^1^, Haoxiang Chen^1^, Chengyu Feng^1^, Fengdi Yang^1^, Chao Zhang^1,3^*, Qing Peng^4^*, Xiaolong Cao^2,3^*

^1^Department of Oncology, Zhujiang Hospital, Southern Medical University, Guangzhou 510282, P.R. China.

^2^Department of Anesthesiology, Zhujiang Hospital, Southern Medical University, Guangzhou 510282, China

^3^Translational Medicine Research Center, Zhujiang Hospital, Southern Medical University, Guangzhou 510282, China

^4^Central Laboratory of The Second Affiliated Hospital, School of Medicine, The Chinese University of Hong Kong, Shenzhen & Longgang District People's Hospital of Shenzhen, Shenzhen, China

^5^Department of Hepatobiliary Surgery II, Zhujiang Hospital, Southern Medical University, Guangzhou 510282, P. R. China.

*Address correspondence to: [zc15996256330@smu.edu.cn](mailto:zc15996256330@smu.edu.cn) (C.Z.); [pengqing@cuhk.edu.cn](mailto:pengqing@cuhk.edu.cn) (Q.P.); [atps@smu.edu.cn](mailto:atps@smu.edu.cn) (X.C.)

^†^These authors contributed equally to this work.

**Fig. S1.** Macroscopic observation of DSH.

**Fig. S2.** Time-sweep rheological analysis of the NK@DSH showing the storage modulus (G’) and loss modulus (G”).

**Fig. S3.** Shear-thinning behavior of NK@DSH.

**Fig. S4.** Macroscopic images showing the extrusion of NK@DSH through a syringe needle.

**Fig. S5.** Quantification of NK cell apoptosis under TGFβ stimulation. Data are presented as the mean ± SD (n = 4, ***p* < 0.01, *****p* < 0.0001).

**Fig. S6.** Quantification of NK cell apoptosis under lactate stimulation. Data are presented as the mean ± SD (n = 4, *****p* < 0.0001).

**Fig. S7.** Quantitative analysis of CD56 immunohistochemical staining in tumor tissues. Data are presented as the mean ± SD (n = 3, ***p* < 0.01).

**Fig. S8.** Quantitative analysis of granzyme B immunohistochemical staining in tumor tissues. Data are presented as the mean ± SD (n = 3, ****p* < 0.001).

**Fig. S9.** Quantitative analysis of IL-2 immunohistochemical staining in tumor tissues. Data are presented as the mean ± SD (n = 3, *****p* < 0.0001).

**Fig. S10.** ELISA analysis of IFN-γ levels in tumor tissues following the indicated treatments. Data are presented as the mean ± SD (n = 3, ***p* < 0.01).

**Fig. S11.** Representative photographs of excised 4T1 tumors following the indicated treatments.

**Table S1.** The sequences of ssDNA (Y1-Y3 and L4-L5) used to synthesize Y-scaffold and L-linker.

|  | DNA sequence (5' to 3') | Calcd MW |
| --- | --- | --- |
| Y1 | TATAGTCTAGAGCCGGGAGCATGAAAAAGATTTGGGATATAGTATAATCATCAT | 15833.38 |
| Y2 | TATAGTCTAGAATGATGATTTATACTATATCCCACCTGACTCCTGGGGAGAAG | 15696.26 |
| Y3 | TATAGTCTAGACTTCTCCCAGGAGTCAGGTGCAATCTTTTTCATGCTCCGGC | 15607.15 |
| L4 | TCTAGACTATAACTAGATACATACAG | 7946.27 |
| L5 | TCTAGACTATACTGTATGTATCTAGT | 7950.25 |
